# Enhancement of prominent texture cues in fly optic flow processing

**DOI:** 10.3389/fncir.2012.00078

**Published:** 2012-10-29

**Authors:** Rafael Kurtz

**Affiliations:** Department of Neurobiology, Bielefeld UniversityBielefeld, Germany

**A commentary on**

**Temporal and spatial adaptation of transient responses to local features**

by O'Carroll, DC., Barnett, P., and Nordström, K. (2012). Front. Neural Circuits 6:74. doi: 10.3389/fncir.2012.00074

During locomotion in a natural environment the visual system of an animal is usually confronted with a large variety of different structures, which cause image motion on the retina. This image motion, called optic flow, is essential for the guidance and calibration of locomotor behavior (Egelhaaf and Kern, 2002; Britten, 2008; Borst et al., 2010). The optic flow produced by distinct, prominent texture elements might be particularly useful for optomotor control. For example, straight horizontal edges present a valuable cue to maintain a constant height above the ground during flight. Accordingly, flies have been found to make use of these cues for visual altitude control (Straw et al., 2010). However, in a cluttered environment the cues that are useful for a particular optomotor task might often be interspersed with many other, less valuable texture elements. An efficient strategy of the neural system to cope with the complexity of optic flow is to extract the structures that provide the most reliable cues. Such a filtering of information would result into strong neuronal responses to distinct structures, while responses to the other, less useful texture elements would be attenuated.

The recent study by O'Carroll et al. (2012) demonstrates that fast contrast gain adaptation in motion vision may help to reduce the complexity of a visual scene by picking out the most prominent texture cues. In direction-selective neurons of the hoverfly brain the authors compared the response elicited by small texture elements of different contrast. They showed that motion of a low-contrast texture, which elicits a sizable neuronal response when presented on its own, causes almost no response when it immediately follows a high-contrast texture. This adaptation effect vanishes when the texture elements are presented in reverse order, with the low-contrast texture leading the high-contrast texture, ruling out that the phenomenon is based on a spatial interaction alone, such as center-surround antagonism.

The neuronal accentuation of high-contrast textures in moving scenery observed by O'Carroll and colleagues is linked to an effect reported by the authors in an earlier study on the same type of horizontal-motion-sensitive neurons, the HS cells (O'Carroll et al., 2011). To examine the effect of individual features within natural settings images of outdoor scenes were presented as if drifting in a horizontal direction behind a narrow, vertically elongated aperture. The image features that were most effective to stimulate the neurons were prominent vertical contrast boundaries, such as tree trunks. However, in series of such contrast boundaries a strong response was mainly observed for the first feature, but not for the subsequent ones.

Previous studies on fly motion-sensitive neurons have already shown that the response to prominent objects, or discontinuities within steady optic flow, is emphasized by adaptation (Liang et al., 2008, 2011; Kurtz et al., 2009). New issues demonstrated by O'Carroll and colleagues are the rapid nature of the adaptation process underlying such feature extraction, and the fact that it is associated with selective contrast gain reduction for less prominent structures. That adaptation influences the neuronal response to image features in a very specific way is also indicated by another interesting aspect of their results. Rather than affecting any low-contrast texture that follows a high-contrast texture, the contrast gain reduction is only activated by continuous vertical line elements. This finding suggests that the effect is located postsynaptic to the computation of orientation selectivity. Nevertheless, it must still operate on a fairly local basis, because line segments corresponding in length to the diameter of the receptive fields of only 2–3 photoreceptors already caused contrast gain reduction. The idea that orientation selectivity is locally computed early in the fly's visual pathway is consistent with the spatial integration properties of inputs to the second visual neuropil, the medulla, reported in an older paper (Arnett, 1972) and with a recent study demonstrating that local, columnar units of the medulla respond in an orientation-selective way (Spalthoff et al., 2012).

According to the established view of fly HS neurons their major function is to provide feedback for the stabilization of a straight flight course and gaze direction, based on balancing any rotational bias in global optic flow (Hausen, 1982a,b; Haag and Borst, 2001). A response enhancement to high-contrast vertical edges relative to low-contrast textures might make sense in this context, because the motion of prominent edges, for example tree trunks, is more likely to be robustly linked to self-motion than the motion of low-contrast textures, for example leafs, which might also tremble in the wind. On the other hand, any mechanism that contributes to the extraction of particular, prominent features increases the pattern dependence of the response (Meyer et al., 2011; O'Carroll et al., 2011), and might therefore contradict a role of HS neurons as pure sensors of global optic flow. Rather, the presence of such mechanisms suggests that these neurons contribute to the acquisition of information about the three-dimensional layout of the environment. During forward flight, the velocity of object motion relative to that of the more distant background is an important cue to coordinate locomotor behavior, for example evasive maneuvers or landing. Responses of HS cells to high-contrast objects located close to the flight trajectory were shown to be enhanced by adaptation when optic flow as encountered by the fly during free flight was used for stimulation (Liang et al., 2008, 2011). This finding emphasizes that such object responses are functionally significant in a natural context. What role the rapid, local contrast-gain adaptation described in the study by O'Carroll and colleagues plays in the processing of complex optic flow and which natural texture elements are accentuated by this type of adaptation is still unclear. These important issues may in the future be tested using optic flow patterns that closely resemble those encountered during natural locomotor behavior.
